# Higher hemoglobin levels are an independent risk factor for adverse metabolism and higher mortality in a 20-year follow-up

**DOI:** 10.1038/s41598-021-99217-9

**Published:** 2021-10-07

**Authors:** Joona Tapio, Hannu Vähänikkilä, Y. Antero Kesäniemi, Olavi Ukkola, Peppi Koivunen

**Affiliations:** 1 Biocenter Oulu and Faculty of Biochemistry and Molecular Medicine, Oulu Center for Cell-Matrix Research, University of Oulu, P.O. Box 5400, 90014 Oulu, Finland; 2 Northern Finland Birth Cohorts, Arctic Biobank, Infrastructure for Population Studies, Faculty of Medicine, University of Oulu, 90014 Oulu, Finland; 3 Medical Research Center Oulu, Faculty of Medicine, Oulu University Hospital and Research Unit of Internal Medicine, University of Oulu, P.O. Box 5000, 90014 Oulu, Finland

**Keywords:** Medical research, Risk factors

## Abstract

The aim of this study was to cross-sectionally and longitudinally examine whether higher hemoglobin (Hb) levels within the normal variation associate with key components of metabolic syndrome and total and cardiovascular mortality. The study included 967 Finnish subjects (age 40–59 years) followed for ≥ 20 years. The focus was on Hb levels, cardiovascular diseases (CVDs) and mortality rates. Higher Hb levels associated positively with key anthropometric and metabolic parameters at baseline. At the follow-up similar associations were seen in men. The highest Hb quartile showed higher leptin levels and lower adiponectin levels at baseline and follow-up (*p* < 0.05) and lower plasma ghrelin levels at baseline (*p* < 0.05). Higher baseline Hb levels associated independently with prevalence of type 2 diabetes at follow-up (*p* < 0.01). The highest Hb quartile associated with higher serum alanine aminotransferase levels (*p* < 0.001) and independently with increased risk for liver fat accumulation (OR 1.63 [1.03; 2.57]) at baseline. The highest Hb quartile showed increased risk for total (HR = 1.48 [1.01; 2.16]) and CVD-related mortality (HR = 2.08 [1.01; 4.29]). Higher Hb levels associated with an adverse metabolic profile, increased prevalence of key components of metabolic syndrome and higher risk for CVD-related and total mortality.

## Introduction

Metabolic syndrome (MetS) is a global epidemic which coincides with the increasing prevalence of obesity. MetS is defined as a group of metabolic disorders which increases the risk for type 2 diabetes (T2DM), cardiovascular diseases (CVD) and total mortality. The major components of MetS are visceral obesity, dyslipidemia and hypertension^1,2^. Non-alcoholic fatty liver disease (NAFLD) is also considered a co-morbidity of MetS^3^. The average prevalence of MetS was 31% in 2017^4^. Individuals with MetS have a 46% increased risk of mortality compared to individuals without the syndrome^5^.

Hb is the main carrier of oxygen in the cardiovascular system. Hb levels are regulated genetically and environmentally and they vary by sex, race, age and living altitude^6,7^. However, individual Hb levels during adult life are relatively stable although Hb levels successively decrease with ageing in men and postmenopausal women^6^. In general, high-end Hb levels within the normal range are considered beneficial for health^6^. However, Hb levels can be elevated by factors such as smoking, a well-known risk factor for metabolic diseases and higher Hb levels are also observed in obesity, which likewise is a well-known risk factor for cardiometabolic diseases^1,2^.

Previous studies with selected cohorts, cross-sectional setting and often including only one sex have shown associations of higher Hb levels with individual components of MetS including insulin resistance, NAFLD, dyslipidemia and hypertension^8–15^. However, higher Hb levels have also been associated with lower glycated hemoglobin (HbA1c) levels^16^. Regarding obesity-related peptide hormones, Hb levels have previously been both positively and negatively associated with serum leptin levels and negatively associated with serum adiponectin levels^17–19^. Studies have also reported lower Hb levels (especially anemic) and very high Hb levels as predictors of total and CVD-related mortality^20–23^. The underlying mechanisms of the reported associations between higher Hb levels and metabolic markers have been poorly understood. Hyperviscosity or changes in plasma volume^10–12,22,24^, endothelial cell dysfunction ^10^ or higher iron/ferritin levels ^13,15^ have been suggested as mediators of these associations. However, we have recently shown that lower Hb levels associate with an overall healthier metabolic profile in males and females in two middle-aged Finnish sea level birth cohorts studied in a longitudinal setting until age of 46 years, and that these alterations are mediated by hypoxia^25^. All in all, the role of Hb levels as a risk factor of MetS and its comorbidities requires further studies.

The aims of this study were (1) to assess cross-sectionally the associations between Hb levels and some 20 key metabolic parameters, obesity-related peptides and ambulatory blood pressure (ABP) measurements in middle-age (average age 51 years) and senescence (average age 72 years), (2) evaluate in a cross-sectional design Hb levels as a risk factor for fatty liver disease, and in a longitudinal design evaluate the role of Hb levels (3) in prediction of the development of impaired glucose metabolism and (4) as a risk factor for CVD events and -related mortality and total mortality. Long-term studies considering the risk profile of the highly prevalent metabolic disorders are needed and this study offers a follow-up period of ~ 20 years, to our knowledge the longest in the literature. Increased information about the risk profile of MetS would eventually lead to decreased morbidity and mortality through improved primary prevention.

## Materials and methods

### Study population

OPERA (Oulu Project Elucidating Risk of Atherosclerosis) is an epidemiological cohort study designed to address the risk factors and clinical endpoints of CVDs. Study subjects were randomly selected between the years 1990 and 1993 as middle-aged, drug-treated hypertensives and their age- and sex-matched control subjects aged 40–59 years (Table S1, Figure S1). Participants were interviewed, examined and tested in our research laboratory. Mortality and hospital events of a total of 1045 subjects were followed up until year 2014. Of the 813 survivors, 600 (62–83 years of age) attended a follow-up examination between 2013 and 2014 (Figure S1).

Inclusion criteria for the current study were Hb levels within Finnish reference values (117–155 g/L for women and 134–167 g/L for men). The Finnish reference values for Hb represent the age-correlated 2.5–97.5% Hb reference range determined by the Finnish National Working Group for basic blood count reference intervals^26^. Of the 1045 subjects, 967 met the inclusion criteria. Of the 967 subjects 558 attended the follow-up. Of the 409 subjects missing the follow-up mortality accounted for 213. Of the 213 deaths 70 were classed as CVD-related. 231 fatal or non-fatal CVD events were recorded during the follow-up (Figure S1).

Study population was first divided to four sex-specific quartiles according to baseline Hb levels (Figure S1). The corresponding sex-specific Hb quartiles were then pooled to form four Hb quartiles (Hb quartile 1–4 where 1 is the lowest and 4 the highest) each of which consisted of the corresponding sex-specific Hb quartiles from both sexes (Figure S1).

The study was conducted according to the principles of the Declaration of Helsinki and approved by the Ethics Committee of the Faculty of Medicine, University of Oulu. Written informed consent was obtained from each participant.

### Clinical measurements

Lifetime smoking burden was determined as pack-years (1 pack-year = 20 cigarettes smoked/day in 1 year) and obtained from a questionnaire. Alcohol consumption was obtained from a questionnaire in number of standard drinks/wk which was converted to g/wk. Physical activity was assessed by a standardized health questionnaire covering physical activity using the method described by Grimby^27^. Physical activity was determined as none, mild, moderate or heavy physical activity. Lipid- and blood pressure medication users were determined from patient records. Lung diseases were classified as asthma, chronic obstructive pulmonary disease or any other lung disease based on anamnesis.

Blood pressure (bp) measurements were undertaken with an automatic oscillometric bp recorder (Dinamap, Critikon Ltd., Ascot, UK) and conducted according to the recommendations of the American Society of Hypertension^28^. Resting bp was measured three times at 1-min intervals from the right arm after the subject had been seated for at least 5 min. The mean value of the second and third bp measurements was used. Ambulatory blood pressure (ABP) measurements were obtained with a non-invasive fully automatic SpaceLabs90207 oscillometric unit (SpaceLabs Inc., Redmond, WA). The measurements were taken every 15 min from 04:00 am to 12:00 pm and every 20 min from 12:00 pm to 04:00 am. The accuracy and reproducibility of bp readings obtained with this device have previously been settled by the British Hypertension Society and the US Association for the Advancement of Medical Instrumentation^29^. The similarity (difference < 5 mmHg) between four SpaceLabs bp measurements and four auscultatory readings using a Y-connector was required to ensure the proper positioning of the cuff. Study subjects were instructed to relax their arm during the measurement. Systolic bp values ≤ 70 mmHg or ≥ 250 mmHg, diastolic bp values ≤ 40 mmHg or ≥ 150 mmHg, and heart rate ≤ 40 or ≥ 150 beats/min were automatically excluded from the analyses. Based on these criteria, less than 3% of the bp measurements were excluded as artefacts^30^.

Body mass index (BMI) was calculated as weight (kg) divided by height squared (m^2^). Height was measured to the nearest centimetre (cm) without shoes using a stadiometer and a sliding horizontal headpiece which was adjusted to rest on the top of the head. Weight was measured to the nearest 0.1 kg with the subject wearing only light underwear without shoes using a SECA personal scale calibrated yearly and used for independent medical weighing and measuring. Waist and hip circumferences were measured using a tape measurer to the nearest 0.1 cm and waist-hip ratio was calculated from these values. All measurements were performed by the same specially trained nurses.

The determination of liver adiposity was based on liver-kidney contrast measured with ultrasonography by one trained radiologist (Markku Päivänsalo) with extensive experience in abdominal ultrasound examinations. The ultrasound examinations were carried out using a Toshiba SSA 270 ultrasound system (Toshiba Corp., Tokyo, Japan) with a scanning frequency of 5 MHz. The entire scanning procedure was captured on video with a Super-VHS video-cassette recorder (Panasonic Corp., Osaka, Japan) and the videotapes were analyzed later. The severity of hepatic steatosis was based on the brightness of the liver in ultrasound scanning and was classified into three groups ranging from 0 to 2 (0 = normal brightness, indicating a non-fatty liver, 1 = medium brightness, a moderate lipid content and 2 = clearly bright, a severe lipid content and fatty liver). Here, we compared subjects with normal brightness of liver (group 0) with those with fatty liver (= combined groups 1 and 2)^31^. Manuscript does not include information or images that could lead to identification of a study participant.

### Laboratory analyses

Blood samples were analysed in NordLab Oulu (former name Oulu University Hospital, Laboratory), a testing laboratory (T113) accredited by Finnish Accreditation Service (FINAS) (EN ISO 15189). Blood Hb levels were determined using the sodium lauryl sulfate method (SLS) and blood leucocytes using the fluorescence flow cytometry method (FFC).

Very-low-density lipoprotein (VLDL) fraction was separated from plasma by ultracentrifugation at 10,500 × *g* for 18 h. Plasma high-density lipoprotein (HDL) cholesterol concentration was measured by mixing 0.5 mL of the VLDL-free fraction with 25 mL of 2.8% (wt/vol) heparin and 25 mL of 2 M manganese chloride and measuring the cholesterol concentration in the supernatant after centrifugation at 1000 × *g* and 4 °C for 30 min. Low-density lipoprotein (LDL) cholesterol concentration was calculated by subtracting the cholesterol concentration in HDL from that in the VLDL-free fraction.

Fasting glucose concentrations were measured with the glucose dehydrogenase method (Diagnostica, Merck, Darmstadt, Germany) and serum insulin levels with a two-site immunoenzymometric assay (AIA-PACK IRI, Tosoh Corp., Tokyo, Japan). HOMA-IR was calculated with the equation (fasting insulin (mU/mL) x fasting glucose (mmol/L) / 22.5). Oral glucose tolerance test (OGTT) was performed in the morning after a 12-h fast immediately after fasting blood had been drawn. Normal glucose tolerance, impaired fasting glucose (IFG), impaired glucose tolerance (IGT) and T2DM were determined according to the World Health Organization (WHO) criteria^32^. Area under the curve (AUC) values for glucose and insulin were determined as integrates from 0 to 2 h time points in OGTT.

High-sensitivity C-reactive protein (hsCRP) was analysed using commercially available enzyme-linked immunosorbent assay (ELISA) kits (Diagnostic Systems Laboratories, Webster, TX) as described before^33^.

Fasting plasma leptin concentration was assessed with a commercial double antibody radioimmunoassay (RIA) (Human Leptin RIA Kit; Linco Research, Inc., St. Charles, MO). Plasma adiponectin concentrations were measured with an ELISA devised in our laboratory, described previously in detail^34^. At baseline the plasma total ghrelin concentration was determined using a commercial RIA kit (Phoenix Pharmaceuticals, Belmont, CA, USA) and at the follow-up using a commercial ELISA Kit (EZGRT-89 K Human Ghrelin ELISA, Merck/MilliporeSigma, Burlington, MO, USA). Correlation between these two methods for determination of total ghrelin concentration has been determined to be very strong^35^.

Alanine aminotransferase (ALT) levels were measured with the recommended method according to the European Committee for Clinical Laboratory Standards as previously described^36^.

### Outcome classification

Information on causes of death and events leading to hospitalization were obtained from the Finnish Causes-of-Death Register and the Hospital Discharge Register. The diagnoses were classified according to the International Classification of Diseases (ICD), Eighth Revision (ICD-8) or Ninth Revision (ICD-9) before 1994 and the Tenth Revision (ICD-10) thereafter. Coronary heart disease (CHD) was defined as diagnoses I20, I21, I22 [ICD-10] and 410, 4110 [ICD-8/9], coronary artery bypass graft or coronary angioplasty as I20–I25, I46, R96, R98 [ICD-10] and 410–414, 798 (not 7980A) [ICD-8/9] as causes of death. CVD was defined as CHD or stroke that included I61, I63 (not I636), I64 [ICD-10] and 431, 4330A, 4331A, 4339A, 4340A, 4341A, 4349A, 436 [ICD-9] or 431 (excluding 43101, 43191), 433, 434, 436 [ICD-8] according to the FINRISK criteria^37^.

### Statistical methods

Continuous variables were presented as mean and categorical variables as percentages. A Pearson correlation coefficient was determined between Hb and metabolic variables to linearly evaluate their associations at baseline and at the follow-up. Differences between Hb quartiles (main explanatory variable) and anthropometric and metabolic variables (outcome) at baseline and follow-up were calculated using general linear model (GLM). To adjust for potential confounding factors, smoking, alcohol consumption and BMI were included as explanatory variables for the Hb quartile comparisons. Statistical significances between the Hb quartiles at baseline and follow-up, and for the development of impaired glucose metabolism were tested using analysis of variance for continuous variables and chi-squared test for categorical variables. A Kruskal–Wallis test was used to determine statistical significance for alcohol consumption and smoking. For impaired glucose metabolism analysis age and sex were additionally included as covariates. A *p* value ≤ 0.05 was considered statistically significant. *p* values < 0.0001 were not given as exact values.

Risk of fatty liver disease according to Hb levels at baseline was estimated with a multivariable logistic regression model. The model included the following covariates: age, sex, BMI, physical activity, alcohol consumption, smoking, LDL cholesterol, HDL cholesterol and lipid medication.

Survival probabilities of total and CVD-related mortality and CVD events were assessed using the Kaplan–Meier survival curves for the Hb quartiles and sex-specific Hb quartiles. Statistical significances of the Kaplan–Meier survival curves were calculated using Log-Rank test. Cox proportional hazard model was used to estimate the association between Hb levels and CVD events, CVD-related mortality and total mortality, respectively. The regression models included Hb quartiles, age, sex, smoking, alcohol consumption, BMI, systolic and diastolic bp, bp medication, LDL and HDL cholesterol and lipid medication. Physical activity was not included in the mortality analysis due a large deviation and complete separation in the regression model. A person-year calculation for events per 100 person-years was used to further elaborate the mortality and CVD events data.

Statistical analyses were calculated using IBM SPSS statistics version 25.0 (IBM Corp, Armonk, NY).

## Results

### Association of Hb levels with key anthropometric, metabolic and other cardiovascular risk factors at baseline and follow-up

**Figure 1:**
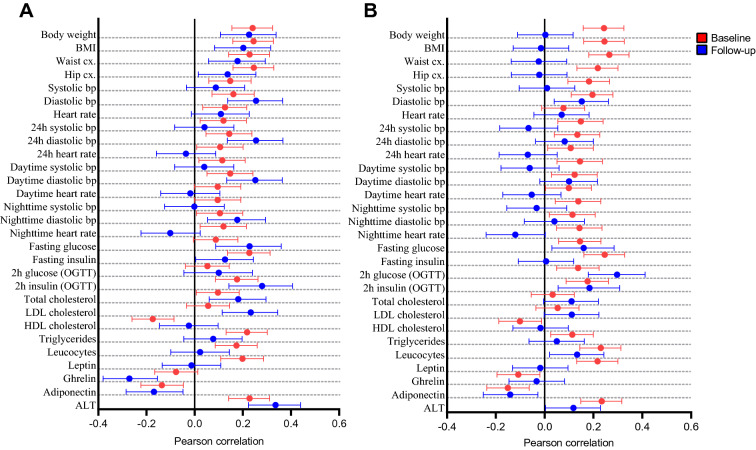
Sex-specific associations of Hb levels with key anthropometric, metabolic and ambulatoric blood pressure (ABP) parameters. Forest plots representing the effect sizes and their 95% confidence intervals for one standard deviation (1-SD) change in key anthropometric measures and metabolic parameters at baseline (red) and at the the 20-year follow-up (blue). (**A**) males, (**B**) females. *BMI* body mass index, *cx* circumference, *bp* blood pressure, *OGTT* oral glucose tolerance test, *LDL* low-density lipoprotein, *HDL* high-density lipoprotein, *ALT* alanine aminotransferase.

First, we examined the association of Hb levels with anthropometric and metabolic parameters in a longitudinal design at two timepoints; baseline at 40–59 years of age, average age 51 years and follow-up at 63–83 years of age, average age 72 years (Fig. 1, Tables S1-S3). At baseline Hb levels associated positively with anthropometric parameters including body weight, BMI and waist and hip circumference in both sexes (Fig. 1). In men these associations remained at the follow-up, but women no longer showed such associations (Fig. 1). For both sexes systolic bp associated positively with Hb levels at baseline but at the follow-up neither sex showed an association (Fig. 1). Diastolic bp associated with Hb levels at baseline in both sexes but at the follow-up the association weakened for women and strengthened for men (Fig. 1). In men heart rate associated with Hb levels at baseline but not at the follow-up (Fig. 1). To amend the resting bp data we used data from ABP monitoring which allows evaluating heart rate and bp continuously. For both sexes Hb levels associated with almost all ABP parameters at baseline (Fig. 1, Tables S2&S3). The positive associations of Hb levels and diastolic ABP were significantly stronger in men at follow-up compared to baseline, but were lost in women, while other associations with ABP were not observed at the follow-up (Fig. 1, Tables S2&S3). The average usage of bp medication increased from 49% at baseline to 78% at the follow-up (Table S1).

Of the key parameters of glucose metabolism, fasting glucose levels associated positively with Hb levels in both sexes at both timepoints (Fig. 1). For both sexes fasting insulin levels associated positively with Hb levels at baseline but the association weakened for men and was lost for women at the follow-up (Fig. 1). Glucose levels 2 h after an OGTT associated positively with Hb levels in women at both time points, strengthening in the follow-up, but no association was seen in men at either timepoint (Fig. 1). Insulin levels 2 h after OGTT associated positively with Hb levels in both sexes at both time points (Fig. 1).

A positive association between Hb levels and total cholesterol levels was observed in men at both time points and but not in women (Fig. 1). For both sexes LDL cholesterol levels associated positively with Hb levels at the follow-up but not at baseline while HDL cholesterol levels associated negatively with Hb levels at baseline in both sexes but not at the follow-up (Fig. 1). For both sexes plasma triglyceride levels associated positively with Hb levels at baseline but not at the follow-up (Fig. 1). At the follow-up 49% of the subjects used lipid-lowering medication compared to 3% at the baseline (Table S1).

Blood leucocyte levels associated positively with Hb levels in both sexes at baseline and this association remained in women but not in men at the follow-up (Fig. 1). For both sexes leptin levels associated positively with Hb levels at baseline but not at the follow-up (Fig. 1). Ghrelin levels associated negatively with Hb levels in women at baseline and in men at the follow-up (Fig. 1). Adiponectin levels associated negatively with Hb levels in men and women at both time points (Fig. 1). For both sexes ALT levels associated positively with Hb levels at both time points (Fig. 1).

### Distribution of cardiovascular risk factors in Hb quartiles at baseline and follow-up

When assessing the distribution of cardiovascular risk factors in Hb quartiles (see Figure S1 for Hb ranges), significant differences in several characteristics, including anthropometric parameters, heart rate, serum lipids, glucose levels, insulin resistance, inflammatory factors, obesity-associated peptide hormones and liver parameters, between the study quartiles were observed at both time points while significant differences in bp and ABP were only observed at baseline (Table 1). The subjects belonging to the highest Hb quartile smoked more, consumed more alcohol, had higher BMI and used bp medication more compared to the other quartiles at both time points (Table 1). The usage of lipid-lowering medication was much more common in all Hb quartiles at the follow-up than baseline, being significantly higher in Hb quartiles 2 and 4 at the follow-up (Table 1). Although the prevalence of lung diseases increased in all Hb quartiles during the follow-up, no difference between the Hb quartiles was observed at either time point (Table 1). The highest Hb quartile at baseline retained the highest mean Hb levels also at the follow-up.

**Table 1:** Characteristics and CVD risk factors of the study-group in the Hb quartiles at baseline and at the follow-up.

Variable	**Baseline**				**Follow-up**			
	Hb quartile 1	Hb quartile 2	Hb quartile 3	Hb quartile 4	Hb quartile 1	Hb quartile 2	Hb quartile 3	Hb quartile 4
Number of patients	264	240	240	223	159	143	147	109
Men (%)	49.2	48.3	48.8	50.7	45	48	46	49
Women (%)	50.8	51.7	51.3	49.3	55	52	54	51
Age (years)	51 (6.1)	51 (5.9)	51 (6.0)	52 (5.9)	72 (6)	72 (5)	72 (5)	72 (5)
Blood pressure medication (%)	49	47	53	61*	74	76	80	86*
Lipid medication (%)	3	2	5	2	48	51	48	50*
Lung disease (%)	12.5	9.6	6.3	8.5	24.5	23.1	25.9	20.2
Hb (g/L)	133 (8.7)	140 (8.4)	146 (8.3)	154 (7.9)*	135 (12.0)	141 (13.1)	142 (16.4)	145 (12.6)*
BMI (kg/m2)	26.7 (4.3)	27.1 (4.4)	27.7 (4.5)	29.5 (4.9)*	28.6 (4.7)	27.8 (4.2)	30.0 (5.8)	30.5 (5.5)*
Smoking (package years)	9 (13.4)	9 (12.4)	8 (13.5)	13 (16.5)*	9 (17.1)	10 (15.2)	9 (17.1)	15 (20.4)*
Alcohol consumption (g/wk)	54 (82.3)	57 (77.2)	60 (91.3)	75 (100.46)*	31 (49)	38 (45)	43 (91)	55 (91)
Waist Circumference (cm)	88 (12.4)	89 (13.7)	90 (12.8)	95 (13.2)*†	95.2 (12.6)	94.3 (13.0)	99.2 (15.3)	100.2 (14.0)*†
WH Ratio	0.86 (0.9)	0.86 (0.9)	0.86 (0.8)	0.88 (0.8)*	0.93 (0.1)	0.94 (0.1)	0.95 (0.1)	0.95 (0.1)
Systolic bp (mmHg)	144 (20.6)	147 (21.3)	150 (21.5)	152 (22.6)*‡	139 (22.6)	137 (22.8)	138 (22.9)	139 (20.3)
Diastolic bp (mmHg)	86 (12.2)	89 (12.1)	90 (11.3)	91 (11.9)*‡	72 (10.0)	72 (9.9)	72 (11.5)	72 (10.4)
Heart rate (bpm)	72 (13.8)	74 (13.2)	74 (13.2)	76 (13.7)*†	67 (11.5)	67 (10.7)	70 (11.8)	71 (12.6)*†
ABP 24 h systolic bp (mmHg)	128 (13.6)	129 (12.6)	131 (13.1)	132 (14.0)*†	133 (13.7)	134 (14.5)	132 (18.2)	131 (15.2)
ABP 24 h diastolic bp (mmHg)	79 (8.3)	81 (7.8)	82 (8.3)	82 (7.8)*‡	72 (7.4)	73 (7.7)	72 (10.8)	71 (8.1)
ABP 24 h heart rate (bpm)	69 (9.7)	70 (9.3)	70 (9.6)	72 (9.7)*‡	65 (9.3)	65 (8.2)	66 (10.1)	65 (10.0)
ABP day systolic bp (mmHg)	132 (16.6)	134 (13.0)	136 (13.8)	137 (14.7)*†	134 (13.8)	135 (14.7)	134 (18.1)	133 (15.2)
ABP day diastolic bp (mmHg)	83 (8.9)	85 (8.0)	86 (8.9)	86 (8.8)*‡	74 (7.5)	74 (7.8)	74 (11.0)	73 (8.1)
ABP day heart rate (bpm)	72 (10.8)	73 (10.0)	73 (10.6)	75 (10.8)*†	66 (9.5)	65 (8.4)	67 (10.4)	67 (8.8)
ABP night systolic bp (mmHg)	115 (14.5)	116 (13.8)	117 (13.3)	119 (14.9)	123 (17.1)	126 (18.0)	125 (21.5)	122 (17.8)
ABP night diastolic bp (mmHg)	69 (9.3)	70 (9.3)	70 (8.4)	71 (9.5)	65 (9.2)	67 (10.1)	66 (12.4)	64 (9.8)
ABP night heart rate (bpm)	61 (8.9)	62 (9.0)	62 (8.8)	64 (8.5)*†	60 (9.5)	61 (8.5)	61 (10.2)	61 (9.0)
Total cholesterol (mmol/L)	5.6 (1.1)	5.8 (1.0)	5.7 (1.0)	5.8 (1.1)	4.9 (1.1)	4.7 (1.1)	4.8 (1.1)	4.6 (0.9)
HDL cholesterol (mmol/L)	1.4 (0.4)	1.4 (0.4)	1.3 (0.4)	1.3 (0.4)*†	1.6 (0.4)	1.5 (0.4)	1.5 (0.4)	1.4 (0.4)*†
LDL cholesterol (mmol/L)	3.4 (0.9)	3.6 (1.0)	3.6 (0.9)	3.6 (1.0)	2.9 (0.9)	2.8 (1.0)	2.9 (1.0)	2.8 (0.9)
Triglycerides (mmol/L)	1.4 (0.9)	1.5 (0.9)	1.6 (1.1)	1.9 (1.1)*‡	1.3 (0.7)	1.3 (0.6)	1.3 (0.6)	1.4 (0.5)
HOMA-IR	2.4 (1.8)	2.7 (3.3)	3.0 (2.8)	4.2 (4.5)*‡	3.6 (2.4)	3.3 (1.8)	3.8 (2.2)	5.1 (3.6)*†
fB-glucose (mmol/L)	4.6 (1.2)	4.6 (1.0)	4.7 (1.2)	5.1 (2.1)*†	5.7 (0.6)	5.7 (0.6)	5.8 (0.5)	6.0 (0.7)*‡
fS-insulin (mU/L)	11.1 (6.6)	12.3 (12.1)	13.8 (10.9)	17.4 (12.3)*‡	13.8 (8.3)	12.7 (6.4)	14.3 (7.8)	18.4 (11.5)*†
2 h glucose (OGTT) (mmol/L)	5.9 (2.9)	5.7 (2.1)	5.9 (2.8)	6.6 (4.0)*†	7.1 (2.4)	6.9 (1.9)	7.1 (2.1)	8.0 (2.6)*‡
2 h insulin (OGTT)	NA	NA	NA	NA	119 (83)	118.0 (90)	125 (90)	147 (93)
AUC glucose	12.4 (5.2)	12.2 (4.2)	12.8 (5.1)	14.6 (6.8)*†	NA	NA	NA	NA
AUC insulin	120 (91)	127 (88)	143 (113)	184 (155)*‡	NA	NA	NA	NA
Leucocytes (E9/L)	5.5 (1.5)	5.8 (1.8)	5.8 (1.6)	6.5 (1.8)*‡	5.9 (1.6)	5.7 (1.3)	5.9 (1.7)	6.6 (3.1)* ‡
hsCRP (mg/L)	3.3 (6.1)	4.2 (9.1)	3.6 (6.0)	3.6 (5.4)	2.9 (5.3)	2.2 (3.3)	3.2 (6.6)	2.8 (3.5)
Leptin (ng/mL)	9.5 (6.9)	10.0 (7.2)	10.4 (7.8)	12.8 (9.7)*‡	22.5 (24.5)	18.0 (15.2)	24.2 (25.3)	25.8 (24.5)*†
Ghrelin (pg/mL)	685 (232)	674 (246)	679 (241)	620 (240)*†	504 (288)	467 (270)	482 (319)	421 (281)
Adiponectin (ug/mL)	16.7 (6.6)	16.6 (7.5)	15.6 (6.2)	14.4 (6.6)*†	16.7 (7.4)	16.2 (7.6)	14.5 (7.6)	13.7 (6.5)*†
ALT (U/I)	27.7 (16.0)	28.4 (15.3)	31.7 (17.6)	40.4 (34.6)*‡	24.3 (10.1)	26.5 (15.0)	27.7 (15.8)	29.1 (17.1)*

The values are mean with (SD) or percentages.

*SD* Standard Deviation, *Hb* Hemoglobin, *BMI* Body Mass Index, *Bp* Blood Pressure, *ABP* Ambulatoric Blood Pressure, *HDL* High-Density Lipoprotein, *LDL* Low-Density Lipoprotein, *HOMA-IR* Homeostatic Model Assessment For Insulin Resistance, *OGTT* Oral Glucose Tolerance Test, *AUC* Area Under The Curve, *hsCRP* high-sensitivity C-reactive protein, *ALT* alanine aminotransferase.

* Statistical significance between the quartiles.

^†^Smoking and alcohol consumption adjusted.

^‡^Smoking, alcohol consumption and BMI adjusted.

When smoking and alcohol consumption were used as covariates, waist circumference, heart rate, HDL cholesterol, HOMA-IR, fasting glucose and -insulin levels, 2 h glucose levels in OGTT, leucocytes and leptin and adiponectin levels differed significantly between the Hb quartiles at both time points, the highest Hb quartile always having the most adverse values (Table 1). When a further adjustment by BMI was done only the difference in leucocytes between the Hb quartiles remained significant at both time points (Table 1). At baseline the differences in systolic and diastolic bp, diastolic ABP and heart rate, serum triglycerides and ALT levels between the Hb quartiles remained after adjustments for smoking, alcohol consumption and BMI, and at the follow-up those for fasting glucose levels and 2 h glucose levels in OGTT (Table 1).

### Association of Hb levels with fatty liver disease in a multivariate risk model

**Figure 2:**
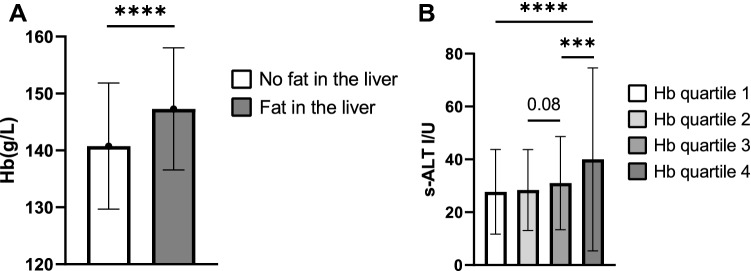
Association of Hb levels with fatty liver disease. (**A**) Hb levels of the subjects with no liver fat accumulation (n = 697) and subjects with liver fat accumulation (n = 256) at baseline. (**B**) Baseline mean ± SD of serum alanine aminotransferase (s-ALT) levels for the corresponding Hb quartiles. Hb quartile 1 has the lowest Hb and Hb quartile 4 the highest Hb levels. For S-ALT analysis was adjusted for smoking, alcohol consumption and BMI. *Hb* hemoglobin.

The highest Hb level quartile showed increased risk for liver fat accumulation in a multivariable logistic regression model after adjusting for covariates (Table 2). At baseline the Hb levels of the subjects with fat accumulation in the liver (n = 256) were significantly higher than those with no fat in the liver (n = 697) (Fig. 2A, Table S4) and the highest Hb level quartile showed higher serum ALT levels after adjusting for covariates (Table 1, Fig. 2B).

**Table 2:** Multivariable logistic regression risk model of liver fat accumulation.

	OR (95% CI)
**Hb (g/L)**	
Hb quartile 1 (low, reference group)	1
Hb quartile 2	0.61 (0.37; 1.02)
Hb quartile 3	1.14 (0.72; 1.81)
Hb quartile 4 (high)	1.63 (1.03; 2.57)
Age (years)	1.031 (1.001; 1.062)
Sex	1.29 (0.85; 1.96)
Smoking (package years)	1.00 (0.99; 1.01)
Alcohol consumption (g/wk)	1.003 (1.001; 1.005)
BMI (kg/m^2^)	1.22 (1.17; 1.27)
Physical activity (none)	0.41 (0.08; 2.20)
Physical activity (irregular)	0.58 (0.12; 2.71)
Physical activity (2x/wk)	0.45 (0.10; 2.71)
Physical activity (≥ 3x/wk)	0.38 (0.08; 1.80)
LDL cholesterol (mmol/L)	1.06 (0.88; 1.27)
HDL cholesterol (mmol/L)	0.39 (0.22; 0.68)
Lipid medication	1.18 (0.46; 3.02)

*OR* Odds Ratio with 95% Confidence Intervals (CIs). The regression model included all variables in the table.

### Association of baseline Hb levels with development of IFG, IGT and T2DM among normoglycemic subjects during the follow-up

**Figure 3:**
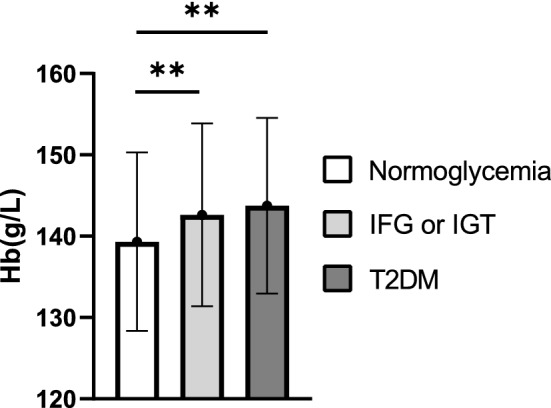
Prevalence of glucose intolerance/diabetes according to baseline Hb levels in previously normoglycemic subjects at the follow-up. Baseline mean ± SD Hb levels of 437 normoglycemic subjects shown in respect to glucose tolerance status at the follow-up. Normal glucose tolerance (normoglycemia), impaired fasting glucose (IFG) or impaired glucose tolerance (IGT) and type 2 diabetes (T2DM) were determined according to the World Health Organization (WHO) criteria. Alcohol consumption, smoking, BMI, sex and age were included as covariates. *Hb* hemoglobin.

We next assessed association of baseline Hb levels in normoglycemic subjects (n = 437) with the prevalence of IFG or IGT and T2DM at the 20-year follow-up (Fig. 3). The mean Hb levels had remained unchanged during the follow-up (Table S5). Mean baseline Hb levels were higher in subjects that developed IGF or IGT (n = 129) and subjects that developed T2DM (n = 98) during the follow-up compared to those who did not (Fig. 3). The highest baseline Hb levels were observed in those who developed T2DM during the follow-up after adjusting for covariates (Fig. 3, Table S5).

### Survival rate analysis of CVD events and CVD-related and total mortality among the Hb quartiles

**Figure 4:**
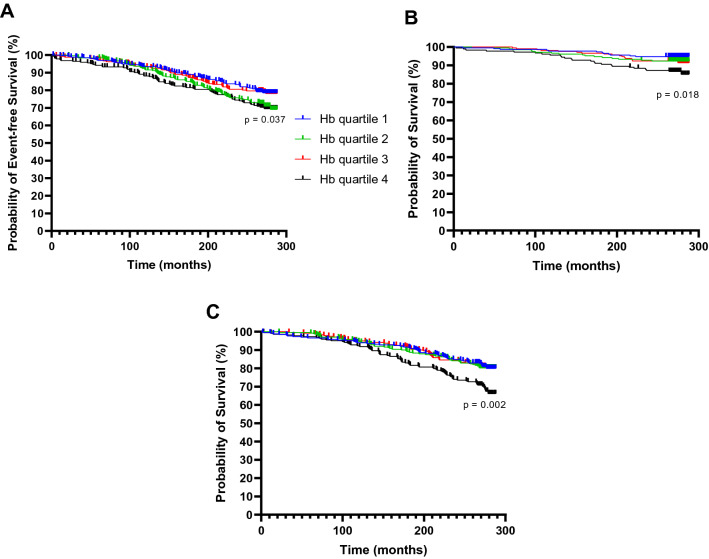
Survival probability according to Hb quartiles. Kaplan–Meier graphs representing survival probabilities for CVD events (n = 231, 23.8%), CVD-related mortality (n = 70, 7.2%) and total mortality (n = 213, 22%) and in each Hb quartile. Hb quartile 1 has the lowest and Hb quartile 4 the highest Hb levels. (**A**) CVD events, (**B**) CVD-related mortality, (**C**) Total mortality. *Hb* hemoglobin.

CVD events (n = 231, 23.8%), CVD-related mortality (n = 70, 7.2%) and total mortality (n = 213, 22%) were assessed with the Kaplan–Meier estimate survival analysis (Fig. 4). The highest Hb quartile had the highest risk for CVD events and CVD-related mortality (Fig. 4A,B). Interestingly, Hb quartile 2 had a similar CVD event-free survival probability to Hb quartile 4 while Hb quartiles 1 and 3 had significantly higher event-free probabilities (Fig. 4A). The highest Hb quartile also showed increased risk for total mortality while Hb quartiles 1–3 all had lower and similar survival probabilities (Fig. 4C). In sex-specific analyses, Hb quartile 4 in women had the lowest survival probability of all outcomes while in men it was the case for total and CVD-related mortalities but not for CVD events where Hb quartile 2 had the highest probability (Figure S2). When evaluated as events/100 person-years, total mortality, CVD mortality and CVD events were significantly highest in Hb quartile 4, the difference being more pronounced in women than men (Table S7, Figure S3).

### Hazard ratios (HR) for CVD events, CVD-related mortality and total mortality among the Hb quartiles

HRs for CVD events, CVD-related mortality and total mortality were assessed with Cox regression models after adjusting for covariates. For CVD events there were no statistically significant differences between the Hb quartiles (Table 3, Table S6). For CVD-related and total mortality the highest Hb quartile (Hb quartile 4) showed a significantly higher HR than the other Hb quartiles (Table 3, Table S6).

**Table 3:** Hazard ratios (HR) for cardiovascular disease (CVD) events, CVD mortality and total mortality according to Hb quartiles.

	CVD events	CVD mortality	Total Mortality
	HR (95% CI)	HR (95% CI)	HR (95% CI)
**Hb (g/L)**			
Hb quartile 1 (Low)	1	1	1
Hb quartile 2	1.43 (0.99; 2.06)	1.63 (0.76; 3.51)	1.03 (0.69; 1.54)
Hb quartile 3	0.96 (0.65; 1.42)	1.62 (0.76; 3.46)	1.02 (0.68; 1.53)
Hb quartile 4 (High)	1.25 (0.85; 1.82)	2.08 (1.01; 4.29)	1.48 (1.01; 2.16)
Age (years)	1.06 (1.04; 1.09)	1.07 (1.02; 1.12)	1.09 (1.06; 1.11)
Sex (male)	1.88 (1.33; 2.68)	2.16 (1.12; 4.17)	1.45 (1.01; 2.08)
Smoking (pack-years)	1.013 (1.004; 1.021)	1.029 (1.015; 1.042)	1.022 (1.013; 1.030)
Alcohol consumption (g/week)	1.000 (0.999; 1.002)	1.002 (1.000; 1.004)	1.002 (1.000; 1.003)
BMI (kg/m2)	1.00 (0.96; 1.03)	1.02 (0.97; 1.08)	1.00 (0.98; 1.04)
Systolic blood pressure (mmHg)	1.00 (0.99; 1.02)	1.02 (1.00; 1.03)	1.00 (0.99; 1.01)
Diastolic blood pressure (mmHg)	0.99 (0.97; 1.00)	0.98 (0.95; 1.01)	1.00 (0.98; 1.02)
Blood pressure medication	1.80 (1.34; 2.41)	1.56 (0.91; 2.67)	1.22 (0.91; 1.63)
HDL cholesterol (mmol/L)	0.60 (0.37; 0.97)	1.37 (0.61; 3.06)	1.12 (0.74; 1.73)
LDL cholesterol (mmol/L)	1.17 (1.01; 1.36)	1.10 (0.84; 1.45)	0.95 (0.81; 1.10)
Lipid medication	1.09 (0.56; 2.10)	1.96 (0.75; 5.14)	1.49 (0.78; 2.87)

Cox regression models representing HR with 95% confidence intervals (CIs) for CVD events (n = 231, 23.8%), CVD-related mortality (n = 70, 7.2%) and total mortality (n = 213, 22%). The regression models include variables as reported in the table. Hb quartile 1 = reference group.

## Discussion

This long-term follow-up study presents higher Hb levels within Finnish reference values (117–155 g/L for women and 134–167 g/L for men) as an independent risk factor for deleterious cardiometabolic health and total and cardiovascular mortality.

We recently studied hypothesis-driven whether normal variation of Hb levels could be used as a surrogate measure for hypoxia^25^. We hypothesized that lower Hb levels were hypoxic and could result via hypoxia-inducible factor (HIF) prolyl 4-hydoxylase (HIF-P4H) inhibition to HIF-mediated reprogramming of energy metabolism and better metabolic health, as we had earlier shown for mice with genetic or pharmacologic inhibition of HIF-P4H^38^. We reported in a large (n = 7,175) systemic study in two Finnish birth cohorts studied until age of 46-years at cross-sectional and longitudinal design associations between Hb levels and many metabolic parameters^25^. Lower Hb levels associated with lower BMI, better glucose tolerance and other metabolic profiles, lesser inflammatory load and lower cholesterol and bp levels and similar associations were found for hematocrit levels and red blood cell counts with BMI^25^. The studied metabolic parameters associated with the expression on HIF target genes, such as glucose transporters, and suggest that Hb levels via regulation of tissue oxygenation can regulate energy metabolism via the HIF pathway, lower Hb levels being beneficial^25^. The findings reported here are in line and extend previous results by us^25^ and others.

The average Hb levels remained stable during the 20-year follow-up period in women and only declined 4 g/L (2.6%) in men. Associations with higher Hb levels and an adverse metabolic profile were observed in both sexes at baseline in average of 51-years suggesting that the individuals with the higher Hb levels possessed glucose intolerance, dyslipidaemia, hypertension, elevated heart rate and a higher inflammatory load. Most of these associations remained but weakened at the 20-year follow-up at average age of 72-years. The decline may have accounted from the increased usage of bp and lipid-lowering medication at the follow-up and by the increased mortality in the highest Hb quartile but likely other factors such as women becoming menopausal during the study contributed, too. In quartile comparisons the adverse metabolic profile was the most prominent in the highest Hb quartile mostly independent of smoking, alcohol consumption and in some cases of BMI. To extend the results we have previously reported in two cohorts studied until middle-age^25^, we show here that the association of Hb levels with an adverse metabolic profile is also observed in senescence. As an example, of the four Hb quartiles studied here, the highest Hb quartile was the only one that classified^39^ as obese by having an average BMI of 30.5 kg/m^2^ at the follow-up. High BMI is a major risk factor CVDs, musculoskeletal disorders (such as osteoarthritis) and some cancers (including breast, prostate and colon cancer)^39^.

Several associations between Hb levels and obesity-related peptide hormones were seen here.

Higher leptin levels and lower plasma ghrelin and adiponectin levels, a pattern well-established to be associated with obesity^40,41^, were observed in the highest Hb quartile at both time points. At baseline all differences were independent of smoking and alcohol consumption and in case of leptin levels, of BMI. At the follow-up the differences between Hb levels and leptin and adiponectin levels, respectively, were independent of smoking and alcohol consumption. Interestingly, the average leptin levels were significantly higher and those of ghrelin lower at the follow-up compared to baseline whereas adiponectin levels showed no obvious change. These results are most likely accounted by weight gain during the follow-up. In women this was probably also influenced by menopause as it increases leptin levels^42^ and lowers ghrelin levels^43^ by altering the levels of sex hormones but does not influence adiponectin levels^44^. To our knowledge this is the first study to report associations between Hb levels and serum ghrelin levels in adults while for leptin levels the previous reports have been conflicting our data agreeing with those in a Swedish senescent men but not middle-aged Japanese men^18,19^.

Several associations between Hb levels and ABP measurements were observed at baseline independent of covariates. Interestingly, many associations, most prominently those for systolic bp, disappeared at the follow-up. In sex-specific analysis a positive association between Hb levels and diastolic APB was observed at both time points in men while in women this was seen at both time points only when evaluated from the office bp measurements. This could reflect the treatment guidelines of the 1990’s where the focus was near-exclusively on treatment of systolic bp^45,46^. In addition, the usage of bp medication increased from 48 to 78% during the follow-up which may account for the loss of these associations. In addition, women are generally more aware of their condition and usually more committed to drug treatment than men^47^, which could explain the sex-specific differences observed. Incremental increases in 24 h ABP (seen here with increase in Hb levels at baseline) have been associated with greater risks of total and CVD-related mortality^48^. Although the increase in bp seen here was minor, it is evident that clustering of multiple cardiovascular risk factors increases morbidity of diseases such as coronary heart disease and consequent mortality^49^.

Higher Hb levels also associated with higher serum ALT levels and risk for liver fat accumulation after adjusting for covariates suggesting that higher Hb levels are an independent risk factor for fatty liver diseases. In addition, the highest Hb quartile was the only quartile with a mean s-ALT exceeding the previously reported thresholds for an increased risk for nonalcoholic steatohepatitis (> 35 U/I) and CVD-related mortality (> 40 U/I)^50,51^. Higher baseline Hb levels were also observed in previously normoglycemic subjects that had IFG, IGT or T2DM at the follow-up after adjusting for covariates, indicating that higher Hb levels could also be a risk factor for the development of T2DM. Furthermore, the highest Hb quartile was the only quartile at the follow-up having a mean 2 h glucose level in OGTT that exceeded the threshold of IGT or IFG set by the WHO (7.8 mmol/L).

The highest Hb quartile also had the lowest survival probability regarding total and CVD-related mortality. This could be the consequence of the positive associations of Hb levels with the key CVD predisposing parameters shown here. Furthermore, since only Hb values within the normal range were included in the analyses the HRs are most likely not affected by abnormally low or high Hb levels-related conditions, such as anaemia or thrombosis, respectively.

The outcomes of these data are in line with our previous data from mice. Mice that have chronically active HIF response due to deficiency in the key HIF-P4H isoenzyme, HIF-P4H-2, or wild-type mice treated with a small molecule HIF-P4H inhibitor, are leaner, have less adiposity and adipose tissue inflammation, they are protected against fatty liver disease of all aetiology and have better glucose tolerance^38,52,53^. In addition, these mice present several differences in the primary causes of death and comorbidities compared to wild-type mice, having *e.g.* less inflammation, liver diseases including hepatocellular cancer, and myocardial infarctions^54^. Furthermore, clinical trials for the treatment of renal anemia with novel HIF-P4H inhibitors roxadustat and daprodustat reported lowered serum cholesterol levels and an improved HDL/LDL lipoprotein profile in the subjects treated with the respected HIF-P4H inhibitors^55,56^.The similarities of these outcome with the Hb levels suggest that the protection against metabolic disorders, such as liver fat accumulation, dyslipidaemia and glucose intolerance, could be mediated by activation of the HIF response by lower tissue oxygen levels in the individuals with lower Hb levels and vice versa individuals with higher Hb levels being at risk.

One study limitation is the use of Hb levels as a surrogate measure for oxygen levels without saturation data. Lung diseases, such as chronic obstructive pulmonary disease or asthma could diminish tissue oxygenation via impaired pulmonary ventilation. This could lower Hb saturation which was not measured here. The prevalence of lung diseases however did not significantly differ between the Hb quartiles at either time point suggesting that the observed results were independent of them. Unfortunately, some of the well-known cardiovascular risk factors such as socioeconomic status, family history or nutritional information were not available for the regression analyses. However, the study population is Finnish representing one of the highest developed countries with free education and health care and highly developed welfare system. Although most data were adjusted for multiple covariates, residual confounding effects need to be considered as an explanatory factor. Also, we have not carried out correction for multiple testing. We believe, however that in the studies that are explanatory in nature, like the current one, it is more important to report all significant findings, even if suggestive, to be considered for replication in another study than taking the risk of rejecting the positive results.

In summary, higher Hb levels at middle-age associated extensively with co-morbidities of MetS at senescence proposing that high Hb levels are an additional risk factor of MetS. Although no clear evidence on causality is provided here, the association of Hb levels was seen extensively across the metabolic phenotype independent of key covariates. Altogether, these data suggest that slight tissue hypoxia, for example by lower endogenous Hb levels, mediate many beneficial effects to cardiometabolic health and increases survival.

## Supplementary Information

Supplementary Information.

## Data Availability

All data is provided in the paper.

## Abbreviations

ABP: Ambulatory blood pressure
ALT: Alanine aminotransferase
BP: Blood pressure
BMI: Body mass index
CVD: Cardiovascular disease
Hb: Hemoglobin
HbA1c: Glycated hemoglobin
HDL: High-density lipoprotein
HIF: Hypoxia-inducible factor
HIF-P4H: HIF prolyl 4-hydroxylase
HOMA-IR: Homeostatic model assessment for insulin resistance
IGT: Impaired glucose tolerance
IFG: Impaired fasting glucose
LDL: Low-density lipoprotein
MetS: Metabolic syndrome
NAFLD: Non-alcoholic fatty liver disease
OGTT: Oral glucose tolerance test
OPERA: Oulu project elucidating risk of atherosclerosis
T2DM: Type 2 diabetes mellitus
