# Supplementary material for: Higher hemoglobin levels are an independent risk factor for adverse metabolism and higher mortality in a 20-year follow-up
Source: Sci Rep. 2021 Oct 7;11:19936. doi: 10.1038/s41598-021-99217-9 (PMC8497471; doi:10.1038/s41598-021-99217-9)

SUPPLEMENTAL MATERIAL

Higher hemoglobin levels are an independent risk factor for adverse metabolism and higher mortality in a 20-year follow-up

Joona Tapio[1], Hannu Vähänikkilä[2], Y. Antero Kesäniemi[3], Olavi Ukkola[3,*], Peppi Koivunen[1,*]

[1]Biocenter Oulu and Faculty of Biochemistry and Molecular Medicine, Oulu Center for Cell-Matrix Research, University of Oulu, 90014 Oulu, Finland. 90014 Oulu, Finland.

[2]Northern Finland Birth Cohorts, Arctic Biobank, Infrastructure for Population Studies, Faculty of Medicine, University of Oulu, 90014 Oulu, Finland.

[3]Medical Research Center Oulu, Oulu University Hospital and Research Unit of Internal Medicine, University of Oulu, 90014 Oulu, Finland

*To whom correspondence should be addressed: Peppi Koivunen, MD, PhD, Faculty of Biochemistry and Molecular Medicine, University of Oulu, P.O. Box 5400, FIN-90014 University of Oulu, Oulu, Finland, Email: peppi.koivunen@oulu.fi,Tel. +358294485822 or Olavi Ukkola, MD, PhD, Faculty of Medicine, University of Oulu and Oulu University Hospital, P.O. Box 5000, FIN-90014 University of Oulu, Oulu, Finland, E-mail: olavi.ukkola@oulu.fi, Tel. +35883154920.

Supplemental tables

Table S1. Characteristics of the OPERA study population. Number of participants (N) in the statistical analyses. The numbers indicate mean for the variables used in the analysis. NA = not available. Hb, Hemoglobin; BMI, Body Mass Index; Bp, Blood Pressure; ABP, Ambulatoric Blood Pressure; HDL, High-Density Lipoprotein; LDL, Low-Density Lipoprotein; HOMA-IR, Homeostatic Model Assessment For Insulin Resistance, OGTT, Oral Glucose Tolerance Test; AUC, Area Under The Curve.

		Baseline			Follow-up		
Variable	Units	All subjects	Males	Females	All subjects	Males	Females
N		967	476	491	558	260	298
Physical activity	times per week	1.7	1.7	1.7	NA	NA	NA
Hb	g/L	143	151	135	140	147	135
Age	years	51	51	52	72	72	72
Smoking	package years	10	15	5	11	17	5
Alcohol consumption	g/w	61	96	27	40	59	24
BMI	kg/m^2	27.7	28.0	27.4	29.1	29.0	29.2
Bp medication	%	48.3	51.3	53.2	78.3	80.4	76.5
Lipid medication	%	2.9	3.6	2.2	48.9	50.4	47.7
Lung disease	%	9.3	7.6	11	23.7	23.1	24.2
Waist circumference	cm	90.5	97.6	83.6	97.0	102.3	92.3
Hip circumference	cm	104.4	104.7	104.1	102.9	101.3	104.2
WH ratio	-	0.87	0.93	0.80	0.94	1.00	0.89
Systolic bp	mmHg	148.1	151.2	145.1	138.2	137.2	139.0
Diastolic bp	mmHg	89.1	92.3	85.9	72.3	74.8	70.0
Heart rate	bpm	73.8	72.2	75.3	68.6	68.7	68.5
ABP 24h systolic bp	mmHg	130	131	129	133	133	132
ABP 24h diastolic bp	mmHg	81	83	79	72	74	71
ABP 24h heart rate	bpm	70	69	71	65	64	66
ABP day systolic bp	mmHg	135	136	133	134	135	134
ABP day diastolic bp	mmHg	85	87	83	74	75	72
ABP day heart rate	bpm	73	73	74	66	65	67
ABP night systolic bp	mmHg	117	117	116	124	125	123
ABP night diastolic bp	mmHg	70	72	68	65	68	63
ABP night heart rate	bpm	62	61	63	61	60	61
Total cholesterol	mmol/L	5.7	5.8	5.7	4.8	4.6	4.9
HDL cholesterol	mmol/L	1.4	1.2	1.5	1.5	1.3	1.6
LDL cholesterol	mmol/L	3.5	3.7	3.4	2.9	2.9	2.9
Triglycerides	mmol/L	1.6	1.8	1.4	1.3	1.3	1.3
HOMA-IR	-	3.0	3.6	2.5	3.8	4.2	3.5
fb-Glucose	mmol/L	4.7	4.9	4.6	5.8	5.8	5.7
fs-Insulin	mU/L	13.5	15.5	11.6	17.6	18.2	17.2
2h Glucose (OGTT)	mmol/L	6.0	5.9	6.0	7.2	7.3	7.1
AUC Glucose	-	13.0	13.5	12.5	NA	NA	NA

AUC Insulin	-	141.9	150.1	134.0	NA	NA	NA
Leucocytes	E9/L	5.9	6.1	5.7	5.8	6.1	6.0
hsCRP	mg/L	3.6	3.7	3.6	2.9	2.6	3.1
Leptin	ng/mL	10.6	6.2	14.7	22.4	11.7	31.8
Grelin	pg/mL	665.9	653.2	678.3	472.4	399.1	536.3
Adiponectin	ug/mL	15.9	13.8	17.9	15.4	13.0	17.6
ALT	U/I	31.8	37.0	26.8	26.7	28.4	25.2

Table S2. Effect sizes for association of Hb levels with anthropometric measures, metabolic parameters and APB measurements at baseline and follow-up in males. Number of participants (N) in the statistical analyses, correlations (*r*) in units of one standard deviation (1-SD) change in anthropometric, metabolic and ABP parameters per 1-SD change in Hb, lower and upper limit for 95% confidence intervals (CIL, CIU) and *p* values for the effect size estimate. ABP, Ambulatoric Blood Pressure; BMI, Body Mass Index; Cx, circumference; Bp, Blood Pressure; OGTT, Oral Glucose Tolerance Test; HDL, High-Density Lipoprotein; LDL, Low-Density Lipoprotein; ALT, alanine aminotransferase.

	**Baseline**					**Follow-up**				
Parameter	N	*r*	CIL	CIU	*p*	N	*r*	CIL	CIU	*p*
Weight	476	0.24	0.15	0.32	<0.0001	260	0.23	0.11	0.34	0.0002
BMI	476	0.25	0.16	0.33	<0.0001	260	0.20	0.08	0.32	0.001
Waist cx	476	0.23	0.14	0.31	<0.0001	260	0.18	0.06	0.29	0.004
Hip cx	476	0.25	0.16	0.33	<0.0001	260	0.14	0.02	0.25	0.027
Systolic bp	476	0.15	0.06	0.23	0.001	260	0.09	-0.03	0.21	0.16
Diastolic bp	476	0.16	0.07	0.25	0.0004	260	0.26	0.14	0.37	<0.0001
Heart rate	446	0.13	0.03	0.22	0.01	260	0.11	-0.01	0.23	0.081
ABP 24h systolic bp	406	0.12	0.02	0.21	0.02	254	0.04	-0.08	0.16	0.5
ABP 24h diastolic bp	406	0.14	0.05	0.24	0.004	254	0.26	0.14	0.37	<0.0001
ABP 24h heart rate	406	0.11	0.01	0.20	0.03	254	-0.04	-0.16	0.09	0.57
ABP day systolic bp	406	0.12	0.02	0.21	0.02	254	0.04	-0.08	0.16	0.52
ABP day diastolic bp	406	0.15	0.05	0.24	0.003	254	0.25	0.13	0.36	<0.0001
ABP day heart rate	406	0.10	0.00	0.19	0.052	254	-0.02	-0.14	0.11	0.77
ABP night systolic bp	406	0.10	0.00	0.19	0.052	249	0.00	-0.13	0.12	0.99
ABP night diastolic bp	406	0.11	0.01	0.20	0.03	249	0.18	0.05	0.29	0.005
ABP night heart rate	405	0.12	0.02	0.21	0.02	249	-0.10	-0.22	0.02	0.11
Fasting glucose	446	0.09	0.00	0.18	0.06	187	0.23	0.09	0.36	0.002
Fasting insulin	446	0.23	0.14	0.31	<0.0001	260	0.13	0.00	0.24	0.04
2h glucose (OGTT)	465	0.05	-0.04	0.14	0.25	187	0.10	-0.04	0.24	0.17
2h insulin (OGTT)	464	0.18	0.09	0.26	<0.0001	188	0.28	0.14	0.41	<0.0001
Total cholesterol	476	0.10	0.01	0.19	0.03	260	0.18	0.06	0.30	0.003
Triglycerides	476	0.22	0.13	0.30	<0.0001	260	0.08	-0.04	0.20	0.21
HDL cholesterol	476	-0.17	-0.26	-0.09	<0.0001	260	-0.02	-0.15	0.10	0.70
LDL cholesterol	476	0.06	-0.03	0.15	0.22	260	0.23	0.11	0.34	<0.0001
Leucocytes	476	0.17	0.09	0.26	<0.0001	260	0.02	-0.10	0.14	0.71
Leptin	446	0.20	0.11	0.29	<0.0001	260	-0.01	-0.13	0.11	0.83
Ghrelin	474	-0.08	-0.17	0.01	0.09	260	-0.27	-0.38	-0.15	<0.0001
Adiponectin	475	-0.14	-0.22	-0.05	0.003	260	-0.17	-0.28	-0.05	0.006
ALT	475	0.23	0.14	0.31	<0.0001	260	0.34	0.22	0.44	<0.0001

Table S3. Effect sizes for association of Hb levels with anthropometric measures, metabolic parameters and APB measurements at baseline and follow-up in females. Number of participants (N) in the statistical analyses, correlations (*r*) in units of one standard deviation (1-SD) change in anthropometric, metabolic and ABP parameters per 1-SD change in Hb, lower and upper limit for 95% confidence intervals (CIL, CIU) and *p* values for the effect size estimate. ABP, Ambulatoric Blood Pressure; BMI, Body Mass Index; Cx, circumference; Bp, Blood Pressure; OGTT, Oral Glucose Tolerance Test; HDL, High-Density Lipoprotein; LDL, Low-Density Lipoprotein; ALT, alanine aminotransferase.

**Baseline**						**Follow-up**				
Parameter	N	*r*	CIL	CIU	*p*	N	*r*	CIL	CIU	*p*
Weight	491	0.24	0.16	0.32	<0.0001	298	0.00	-0.11	0.12	0.96
BMI	491	0.25	0.16	0.33	<0.0001	298	-0.02	-0.13	0.10	0.79
Waist cx	489	0.27	0.18	0.35	<0.0001	297	-0.02	-0.14	0.09	0.69
Hip cx	489	0.22	0.13	0.30	<0.0001	298	-0.02	-0.14	0.09	0.70
Systolic bp	491	0.18	0.10	0.27	<0.0001	298	0.01	-0.10	0.12	0.87
Diastolic bp	491	0.20	0.11	0.28	<0.0001	298	0.15	0.04	0.26	0.01
Heart rate	491	0.08	-0.01	0.16	0.09	298	0.07	-0.04	0.18	0.23
ABP 24h systolic bp	427	0,15	0,05	0,24	0,002	270	-0,07	-0,18	0,05	0,28
ABP 24h diastolic bp	427	0,13	0,04	0,23	0,01	270	0,08	-0,04	0,20	0,18
ABP 24h heart rate	427	0,11	0,01	0,20	0,03	270	-0,07	-0,19	0,05	0,26
ABP day systolic bp	427	0,15	0,05	0,24	0,003	270	-0,06	-0,18	0,06	0,32
ABP day diastolic bp	427	0,12	0,03	0,22	0,01	270	0,10	-0,02	0,22	0,10
ABP day heart rate	427	0,10	0,00	0,19	0,04	270	-0,05	-0,17	0,07	0,39
ABP night systolic bp	424	0,14	0,04	0,23	0,004	254	-0,03	-0,16	0,09	0,60
ABP night diastolic bp	424	0,11	0,02	0,21	0,02	254	0,04	-0,08	0,16	0,52
ABP night heart rate	424	0,14	0,05	0,24	0,003	254	-0,12	-0,24	0,00	0,06
Fasting glucose	491	0.15	0.06	0.23	0.001	226	0.16	0.03	0.28	0.02
Fasting insulin	491	0.25	0.16	0.33	<0.0001	298	0.01	-0.11	0.12	0.92
2h glucose (OGTT)	485	0.14	0.05	0.22	0.002	226	0.30	0.17	0.41	<0.0001
2h insulin (OGTT)	482	0.18	0.09	0.26	0.0001	225	0.18	0.05	0.31	0.006
Total cholesterol	491	0.03	-0.06	0.12	0.46	298	0.11	0.00	0.22	0.06
Triglycerides	491	0.11	0.02	0.20	0.01	298	0.05	-0.06	0.16	0.39
HDL cholesterol	491	-0.10	-0.19	-0.01	0.03	298	-0.02	-0.13	0.10	0.78
LDL cholesterol	491	0.05	-0.04	0.14	0.24	298	0.11	0.00	0.22	0.05
Leucocytes	491	0.23	0.14	0.31	<0.0001	298	0.13	0.02	0.24	0.02
Leptin	488	0.22	0.13	0.30	<0.0001	297	-0.02	-0.13	0.10	0.75
Ghrelin	490	-0.11	-0.20	-0.02	0.02	298	-0.03	-0.15	0.08	0.57
Adiponectin	488	-0.15	-0.24	-0.06	0.001	297	-0.14	-0.25	-0.03	0.02
ALT	490	0.23	0.15	0.32	<0.0001	298	0.12	0.00	0.23	0.04

Table S4. Mean hemoglobin (Hb) levels of the subjects with no fat in the liver and subjects with fat in the liver. Number of participants (N) in the statistical analyses, mean Hb levels (g/L), standard deviation (STDEV, g/L), standard error of the mean (SEM, g/L), and *p* values for the difference between the groups. Hb, hemoglobin.

Group	N	Mean Hb	STDEV	SEM	*p*
No fat in the liver	697	140.8	11.1	0.42	
Fat in the liver	256	147.3	10.7	0.67	<0.0001

Table S5. Mean baseline hemoglobin (Hb) levels of normoglycemic subjects according to glucose tolerance status at the 20-year follow-up. Number of participants (N) in the statistical analyses, mean Hb levels (g/L), standard deviation (STDEV, g/L) and adjusted *p* value for the difference between the groups. Alcohol consumption, smoking, BMI, sex and age were included as covariates.

Group	N	Mean Hb	STDEV	*p*
**Baseline**				
Normoglycemic	437	141.3	11.2	
**Follow-up**	437	141.1	14.3	
Normoglycemic (reference group)	210	139.3	11.0	
IGF or IGT	129	142.6	11.2	0.008
Diabetes	98	143.7	10.8	0.001

Table S6. Total deaths, CVD-related deaths and CVD events in each Hb quartile during the 20-year follow-up. Number of participants (N) in the statistical analyses. Hb, hemoglobin; CVD, cardiovascular disease.

	Hb quartile 1	Hb quartile 2	Hb quartile 3	Hb quartile 4	Total
N	227	209	211	181	828
Deaths	50	47	46	70	213
CVD-related deaths	12	16	17	25	70
CVD events	53	66	50	64	233

Table S7. Events recorded per 100 person-years. Mean values for events recorded per 100 person-years in the study. SEM = Standard Error of the Mean, n = Number of participants in the statistical analyses**.**

	**Hb quartile 1**			**Hb quartile 2**			**Hb quartile 3**			**Hb quartile 4**			**All subjects**		
**All**	Mean	SEM	n	Mean	SEM	n	Mean	SEM	n	Mean	SEM	n	Mean	SEM	n
Total mortality	0,89	0,13	264	0,93	0,14	240	0,90	0,13	240	1,56	0,19	223	1,05	0,07	967
CVD mortality	0,21	0,06	264	0,32	0,08	240	0,33	0,08	240	0,56	0,11	223	0,35	0,04	967
CVD events	0,95	0,13	264	1,30	0,16	240	0,96	0,14	240	1,40	0,18	223	1,14	0,07	967
**Men**	Mean	SEM	n	Mean	SEM	n	Mean	SEM	n	Mean	SEM	n	Mean	SEM	n
Total mortality	1,43	0,23	130	1,10	0,21	116	1,17	0,22	117	1,89	0,29	113	1,39	0,12	476
CVD mortality	0,41	0,12	130	0,49	0,14	116	0,44	0,13	117	0,70	0,18	113	0,51	0,07	476
CVD events	1,39	0,23	130	1,91	0,28	116	1,29	0,23	117	1,67	0,27	113	1,56	0,13	476
**Women**	Mean	SEM	n	Mean	SEM	n	Mean	SEM	n	Mean	SEM	n	Mean	SEM	n
Total mortality	0,41	0,12	134	0,76	0,17	124	0,64	0,16	123	1,22	0,23	110	0,73	0,08	491
CVD mortality	0,03	0,03	134	0,15	0,08	124	0,23	0,09	123	0,41	0,14	110	0,19	0,04	491
CVD events	0,55	0,14	134	0,73	0,17	124	0,64	0,16	123	1,13	0,23	110	0,74	0,08	491

Supplemental figures

Figure S1. Flow-chart representing the study population and analyses done. Number of participants (n) in the statistical analyses. OPERA, Oulu Project Elucidating Risk of Atherosclerosis; Hb, hemoglobin; bl, baseline; fu, follow-up; ABP, ambulatory blood pressure; IGT, impaired glucose tolerance; IFG, impaired fasting glucose; T2DM, type 2 diabetes; CVD, cardiovascular disease.

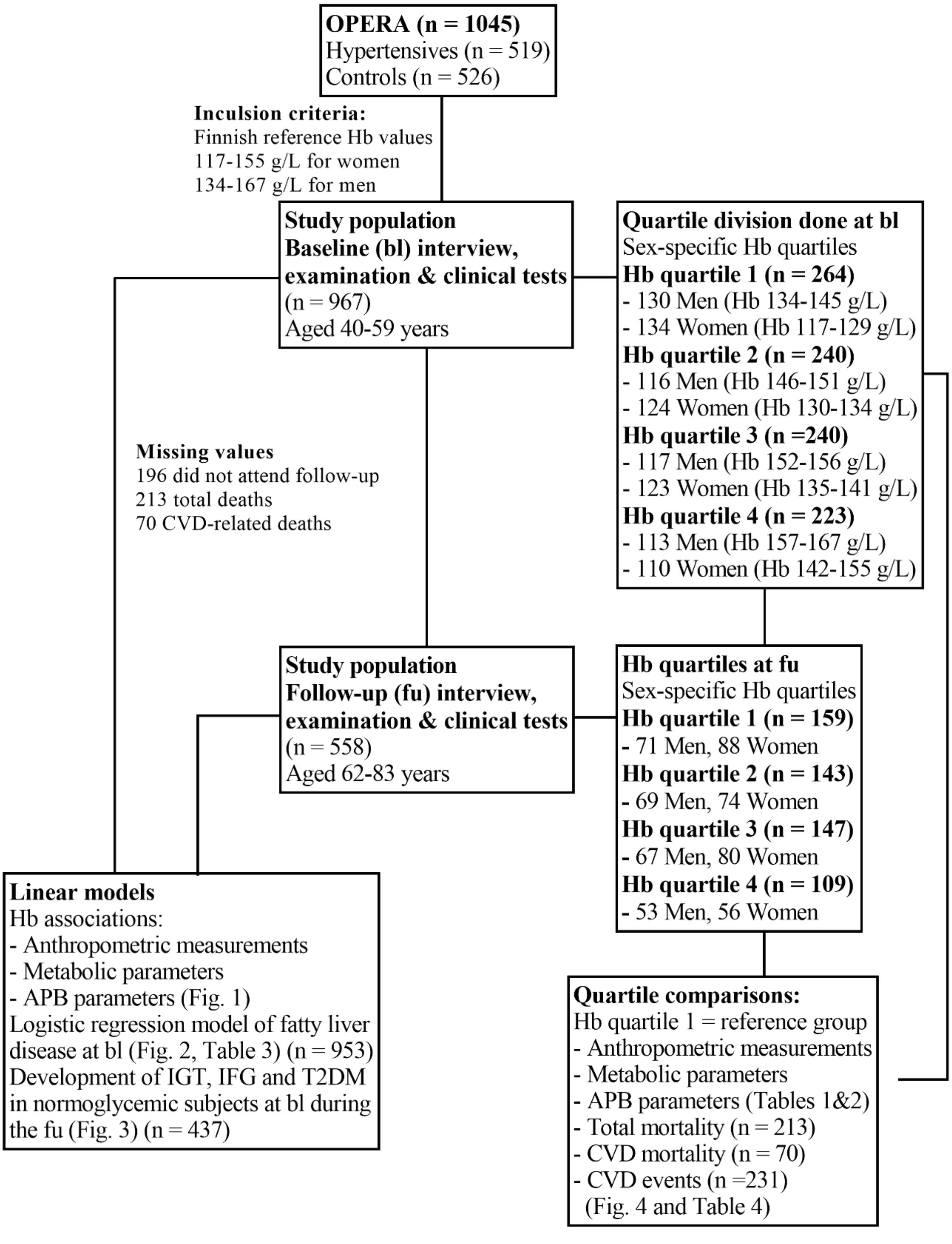

Figure S2. Sex-specific survival probability according to Hb quartiles. Kaplan-Meier graphs representing survival probabilities for CVD events (A = men (n=154), B = women (n=77)), CVD-related mortality (C = men (n=50), D = women(n=20)) and total mortality (E = men (n=137), F = women (n=76)) in each sex-specific Hb quartile. Hb quartile 1 has the lowest and Hb quartile 4 the highest Hb levels. Hb, hemoglobin.

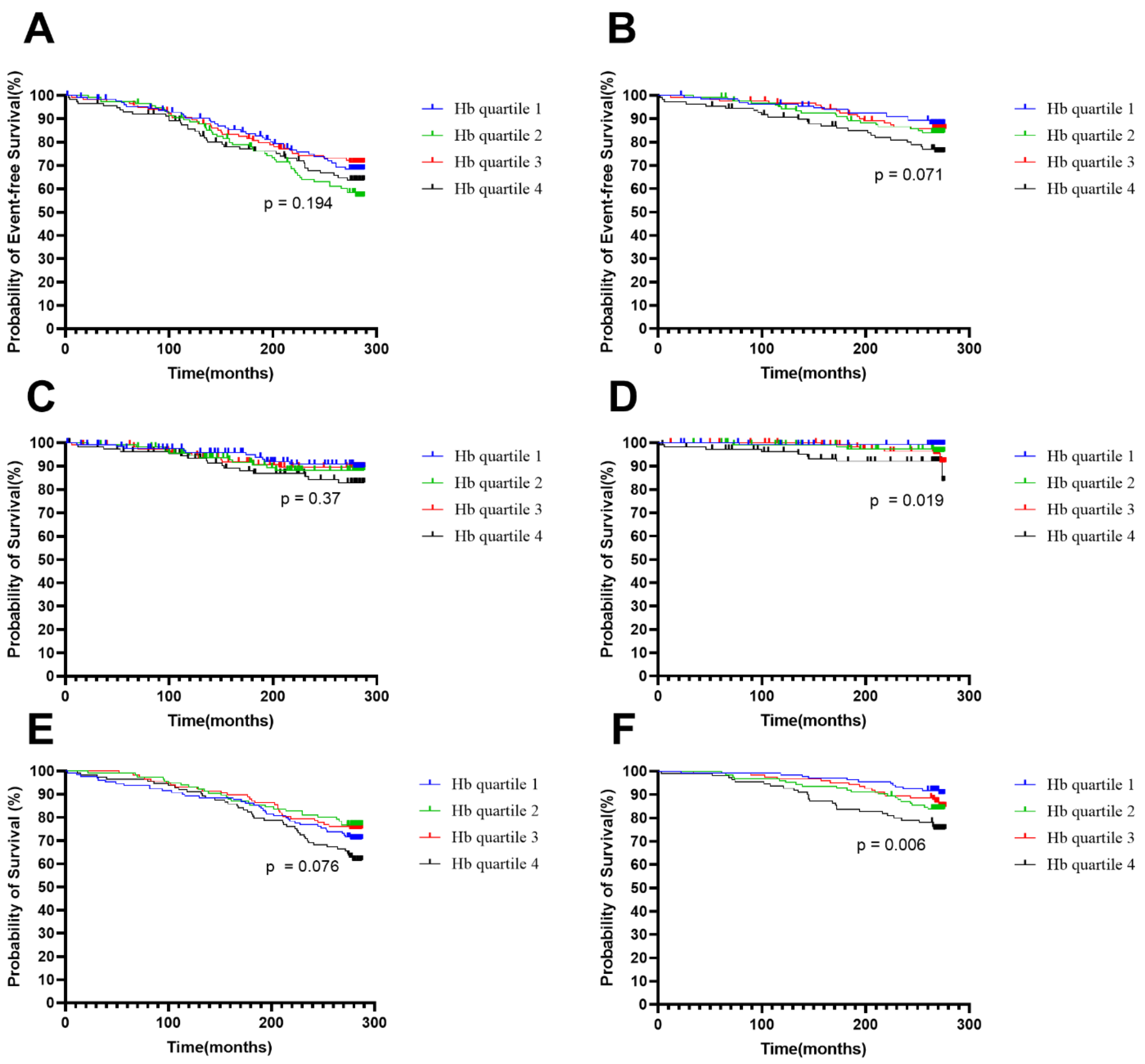

Figure S3. Total mortality, CVD mortality and CVD Events per 100 person-years. A = all subjects, B = men, C = women. Hb = hemoglobin.

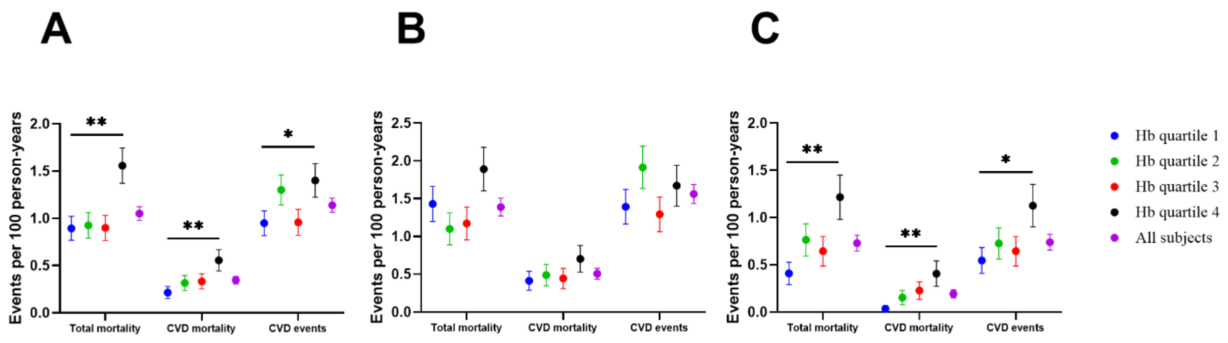

Supplement: Supplementary file 1 — Supplementary Information. [file 41598_2021_99217_MOESM1_ESM.pdf]
